# Imaging the Neutrophil Phagosome and Cytoplasm Using a Ratiometric pH Indicator

**DOI:** 10.3791/55107

**Published:** 2017-04-05

**Authors:** Juliet R. Foote, Adam P. Levine, Philippe Behe, Michael R. Duchen, Anthony W. Segal

**Affiliations:** ^1^Centre for Molecular Medicine, Division of Medicine, University College London; ^2^Cell and Developmental Biology, University College London

**Keywords:** Immunology, Issue 122, phagocytosis, neutrophil, phagosomal pH, ratiometric imaging, fluorescence microscopy, innate immunity

## Abstract

Neutrophils are crucial to host innate defense and, consequently, constitute an important area of medical research. The phagosome, the intracellular compartment where the killing and digestion of engulfed particles take place, is the main arena for neutrophil pathogen killing that requires tight regulation. Phagosomal pH is one aspect that is carefully controlled, in turn regulating antimicrobial protease activity. Many fluorescent pH-sensitive dyes have been used to visualize the phagosomal environment. S-1 has several advantages over other pH-sensitive dyes, including its dual emission spectra, its resistance to photo-bleaching, and its high pKa. Using this method, we have demonstrated that the neutrophil phagosome is unusually alkaline in comparison to other phagocytes. By using different biochemical conjugations of the dye, the phagosome can be delineated from the cytoplasm so that changes in the size and shape of the phagosome can be assessed. This allows for further monitoring of ionic movement.

**Figure:**
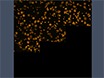


## Introduction

The neutrophil is the most abundant innate immune cell in the body. Its main function consists of patrolling the bloodstream and engulfing and digesting the foreign particles that it may encounter in a process known as phagocytosis^1^,^2^. The particles are degraded in an intracellular compartment called the phagosome. The activation of neutrophil NADPH oxidase, the isoform NOX2, initiates a cascade of biochemical reactions that culminates in the death of the pathogen. NOX2 protein subunits proceed to form an electron transport chain complex in the phagosomal membrane^3^. Once activated, it transports electrons from NADPH across the membrane to molecular oxygen inside the phagosome, producing superoxide anions and further reactive oxygen species. This is known as the respiratory burst, and it is thought to be essential for efficient microbial killing and digestion^2^. However, this exclusive movement of negative charge across the membrane would soon inactivate NOX2 if it were not compensated for by positive charge moving in and/or negative charge moving out of the phagosome. It has been well established that the majority of charge compensation in the neutrophil is carried out by the proton channel HVCN1^4^,^5^. This channel allows for the passive movement of protons down their electrochemical gradient from the cytosol into the phagosome. Proton concentration is reflected by pH, so for a given level of oxidase activity, measuring the pH in the phagosome can provide information on the relative participation of protonic and non-protonic pathways in charge compensation.

The human neutrophil phagosome has an alkaline pH of approximately 8.5 for 20-30 min after phagocytosis^5^. This implies the existence of additional non-proton ion channels in NOX2-induced charge compensation, as the fusion and release of the contents of the acidic granules and sole compensation by HVCN1 would maintain an acidic environment, in contrast to that observed. The movement of ions to compensate this negative charge may also exert changes in phagosome size via osmosis. These may be ions present in the neutrophil at high physiological concentrations: potassium ions have been shown to move into the phagosome^6^, and chloride ionic movement is another candidate important for neutrophil function^7^.

The regulation of pH in the phagosome is vital for antimicrobial protease activity^5^. Myeloperoxidase (MPO) appears to have optimal activity at pH 6, while for cathepsin G and elastase, the optimal levels are pH 7-9 and pH 8-10, respectively^5^. Therefore, transient change in phagosomal pH may provide activity niches for different enzymes to function. Understanding how pH is involved in neutrophil microbial killing may provide useful information for the design of novel neutrophil-augmenting microbial agents.

The neutrophil phagosome is a highly reactive environment. This makes it difficult to accurately assess pH, because dyes may be easily oxidized, leading to technical artefacts. Historically, fluorescein isothiocyanate (FITC) has been the dye of choice to measure intracellular pH^8^,^9^. However, there are some disadvantages for its use in measuring neutrophil phagosomal pH. It has a pKa of 6.4^10^, meaning that it can only accurately be used to assess pH levels from 5 to 7.5^8^, as it saturates at pH < 8^11^. As the neutrophil phagosomal pH can become much more alkaline^5^, FITC cannot capture the full range of potential pH changes. A further significant problem with FITC in the context of neutrophils is that it is thought to be photobleached by MPO^12^. The MPO inhibitor, sodium azide, can be used to limit photobleaching^13^, but it has been shown that sodium azide directly lowers the phagosomal pH in an MPO-independent manner and is thus inappropriate for use in such assays^5^.

Compared to other intracellular dyes, S-1 has a relatively high pKa of 7.5^10^. In acidic conditions, the molecule is protonated and produces an emission signal between 560 and 600 nm when excited at 488 nm or above. When the molecule is deprotonated in more alkaline conditions, the emission wavelength is over 600 nm. A ratio of the fluorescence intensities at these two wavelengths indicates the emission shift, which is more is reliable than single fluorescence measurements, as it is unaffected by fluorophore concentration and cell structure. S-1 can be conjugated to antigenic material, such as zymosan^14^, although heat-killed (HK) *Candida albicans* is preferred, as the larger surface area gives a more consistent fluorescence reading.

We have also used a modification of this method to study temporal changes in pH (**Figure 3**)^5^. This method for measurement of cytosolic pH can be easily applied to other cell types, as described elsewhere^15^,^16^, and cells with more alkaline phagosomes^14^.

## Protocol

Ethics statement: All animal work was conducted with the license and approval of the United Kingdom Home Office. Human participation in this research was approved by the Joint UCL/UCLH Committees on the Ethics of Human Research. All participants provided informed consent.

### 1. Preparation of *C. albicans*

1. Grow a *Candida* disc (see Materials List) on a YPD agar plate as per the manufacturer's instructions. Pick a colony and add it to 15 mL of YPD broth. Incubate it in a shaking incubator at 30 °C and 200 rpm until the broth is cloudy (usually about 2 days, or to a concentration of roughly over 1 x 10^9^/mL).
2. Spin down the *Candida* medium and resuspend it in 50 mL of phosphate-buffered saline (PBS). Centrifuge at 3,000 x g for 10 min. Repeat this step twice.
3. Place the tube containing 50 mL of PBS/*Candida* medium in a water bath preheated to 60 °C so that the entire tube is submerged for 1 h.
  1. To confirm that the *Candida* are heat-killed, streak a sample onto a YPD agar plate and incubate overnight at 30 °C. Adjust the heat-killed (HK) *Candida* concentration to 5-9 x 10^8^/mL, depending on *Candida* growth, and store it in 1 mL aliquots in a -20 °C freezer. NOTE: All cell counts in this protocol were performed using an automated cell counter.

### 2. S-1 Coupling to Heat-killed (HK) *Candida*

1. Prepare an aliquot of carboxy-S-1 succinimidyl ester (50 µg) by diluting it in 100 µL of high-grade dimethyl sulfoxide (DMSO). Vortex well to mix.
2. Prepare 1 mL of 1 x 10^8 ^HK *Candida* in 0.1 M sodium bicarbonate (pH 8.3) in a 15 mL tube.
3. Add 100 µL of carboxy-S-1 one drop at a time to the HK *Candida* while mixing on a vortex at roughly 2,000 rpm (medium to high speed). Wrap aluminum foil around the tube and place it on a roller at room temperature for 1 h.
4. Wash the HK *Candida-*S-1(HKC-S-1) three times by centrifugation at 2,250 x g for 10 min each. For the first two washes, resuspend the pellet in 15 mL of 0.1 M sodium bicarbonate (pH 8.3). After the third wash, resuspend it in 1 mL of balanced salt solution (BSS) buffer (see **Table 1**).
5. Transfer the HKC-S-1 suspension into tubes in 100 µL aliquots and store them at -20 °C. NOTE: Use tubes with low surface binding material, if possible, as HKC-S-1 particles can stick to the wall of normal centrifuge tubes.
6. Opsonize HKC-S-1
  1. For human neutrophils, add 100 µL of human IgG serum to 100 µL of thawed HKC-S-1.
  2. For mouse neutrophils, add 50 µL of normal mouse serum and 50 µL of mouse *Candida* immune serum (generated from C57B6 mice injected with HK *Candida*)^5^ to 100 µL of HKC-S-1.
  3. Mix it on a heat-shaker at 37 °C and 1,100 rpm for 60-90 min, and then on a 4 °C roller for 2 h.
  4. Wash three times in BSS buffer by centrifugation at 17,200 x g for 1 min each. Resuspend in 100 µL of BSS buffer.

### 3. Isolation of Neutrophils

1. For human peripheral blood neutrophils, take 15 mL of blood by venipuncture from a healthy donor and place it into a 20 mL syringe containing 90 µL of 1,000 IU/mL heparin sodium solution (60 µL of heparin/10 mL of blood).
2. Using a pipette tip, inject 1.5 mL of 10% dextran solution into the syringe. Invert it gently 3 times and leave it standing upright on the bench.
3. Wait 30-60 min, until the blood is split roughly in half, with a top buffy coat layer that is straw-colored and a bottom layer containing erythrocytes.
4. Carefully push out the top layer through a needle or remove it with a pipette tip. Put it in a 15 mL tube. Avoid taking out the red layer. Using a 5 mL pipette, add 3-4 mL of density gradient medium (see Materials List) to the bottom of the tube under the buffy coat layer to obtain two distinct layers. Centrifuge at 913 x g for 10 min. NOTE: If using mouse neutrophils (see reference^17^ for mouse neutrophil isolation), use the cell suspension that has been flushed from the bone marrow instead.
5. Pour off the supernatant, leaving behind a red pellet. Gently vortex to disturb the pellet. Add 7 mL of distilled, autoclaved H_2_O to the pellet and invert for 20 s to resuspend the pellet. Add 7 mL of 2x saline and invert a few times to mix, lysing the remaining red blood cells.
6. Centrifuge at 300 x g for 5 min. Pour off the supernatant and resuspend the pellet in BSS buffer to approximately 4 x 10^6^/mL. NOTE: For optimal microscopy of the cells, keep the cell suspension concentration between 1.5-6 x 10^6^/mL.

### 4. Preparation of Slides

1. Pre-treat an 8-well microscopy plate (see Materials List) with 200 µL of poly-L-lysine (0.01% solution) in each well for 40-60 min at room temperature.
2. Remove the poly-L-lysine (can be reused) and wash the wells twice with 200 µL of distilled H_2_O.
3. Add 200 µL of cell suspension prepared in step 3.6 to each well. Incubate at room temperature for 30-60 min.
4. Prepare an aliquot of 5-(and-6)-carboxy S-1 acetoxymethyl (S-1-AM) ester (50 µg) by adding 100 µL of high-grade DMSO to one tube. Vortex to mix. When not in use, store at -20 °C.
5. In a small tube, add 1.7 mL of BSS buffer and 20 µL of S-1-AM. Vortex to mix.
6. Wash the wells twice with 200 µL of BSS buffer, and then replace the buffer with the S-1-AM solution. Take care to not disturb the monolayer of cells attached to the bottom of the well by gently pipetting down the walls of the wells. Incubate at room temperature for at least 25 min.
7. Wash the wells twice with 200 µL of BSS buffer. To test an inhibitor, make up a "master mix" of the appropriate drug in BSS buffer. Wash the wells twice in the inhibitor solution. NOTE: Make sure to use the same amount of drug vehicle (*e.g.,* DMSO or ethanol) in the control well as was used for the inhibitor. If testing the effect of zinc chloride, use BSS buffer lacking KH_2_PO_4_ (the HEPES can buffer the solution alone), as zinc precipitates with phosphate.
8. Sonicate the opsonized HKC-S-1 (section 6.2) for approximately 3 s at 5 amplitude µm. Add 10 µL to each well. Incubate the plate at 37 °C for 15-20 min to allow phagocytosis, making the cells ready to be imaged for snapshots of phagocytosis.

### 5. Confocal Microscopy

1. Using a confocal microscope, adjust the laser wavelength so that the cells are excited at 555 nm and the emission is detected by two channels, or detectors: 560-600 nm and over 600 nm. NOTE: Specific microscope parameters will differ for each microscope and need to be optimized by the researcher.
2. View the cells using a 63X lens with oil. Use a tile-scan image on continuous setting to view the center tile. Using the fluorescence intensity and gain of detector channels, adjust the focus and intensity of the laser and the gain of the two channels to optimize the image.
3. Split the image using the settings to view both channels; check that there is no saturation of fluorescence intensity in the cytoplasm or vacuoles. Saturation appears as red dots. Reduce the laser intensity so that there is a minimal number of red dots, but the cells and phagosomes are bright enough to see clearly.
4. When satisfied, click on "Start experiment." A 9-tile scan image is generated. Save the image as a complex file recommended by the software that contains a composite image of the two channels (not jpeg or tiff).

### 6. Calibration Experiments

1. **Construct the pH standard curve with *Candida *alone.**
  1. Prepare the buffers from pH 3.0 to 13.0, as per **Table 1**.
  2. Take one aliquot (100 µL) of HKC-S-1. Spin it down for 1 min at 17,200 x g. Resuspend it in 500 µL of the assigned buffer.
  3. Starting from the lowest pH range, add the suspension to one pre-treated well (from step 4.2). Place the slide on the confocal microscope on a heated stage set to 37 °C.
  4. Record the fluorescence every min for 10 min. Repeat for each buffer.
2. **Saponin standard curve.**
  1. Isolate 1 x 10^7^ human neutrophils^5^ and resuspend them in 1 mL of the lowest pH buffer.
  2. Follow the method described in section 4 for measuring phagosomal pH in neutrophils.
  3. After incubation with the *Candida* suspension for 20 min, add 0.3% saponin (15 µL of 10% stock to one pre-treated well (from step 4.2)) to the dish, and then incubate for 20 min at 37 °C. Take an image every minute for 10 min. Repeat for each buffer.
3. **Cytosolic pH standard curves using the high K^+^/nigericin technique**^5^,^18^,^19^.
  1. Make up the buffers described in **Table 1**, from pH 4.0 to 13.0.
  2. Follow steps 4.1-4.7, leaving only neutrophils in the pre-treated wells — each well to contain a different buffer. Add the slide to the 37 °C heated stage and record the fluorescence every min for 10 min.
  3. Note: It may take some time for the cytosolic pH to stabilize with the external solution, but after this point, the S-1 ratio value becomes constant. Measuring the extracellular HKC-S-1 in each experiment can serve as a control to check that any inhibitors added do not interfere with the S-1 emitted fluorescence or affect the buffer's pH.

### 7. Image Analysis

NOTE: Here, instructions for image analysis (quantitation of fluorescence) using the free software ImageJ is provided. Use of ImageJ is recommended.

1. Download and install ImageJ version >1.46r as per the developer's instructions (https://imagej.nih.gov/ij/). Install the supplementary code file attached with this protocol called “Phagosomal measurement.”
2. Open Image J and load the image file chosen for analysis onto the toolbar. To combine the two channels, use Image>Color>Make Composite.
3. Right-click to choose a file in which to store results.
4. Click on the line tool on the tool bar and double-click to increase the width to "2."
5. Draw a line across the width of a phagosome, and then right-click to "measure pH". The average intensity of the two channels is acquired, and their ratio is calculated automatically by the code file in ImageJ.
6. After finishing the measurements, right-click to choose "save file."
7. To measure the phagosome area, use the fourth icon along the tool bar to draw free-hand around the area. Right-click to select "measure area." NOTE: The results are saved and a log file is generated in the directory selected. Results may be processed using spreadsheet, R, or an equivalent software. The relationship between fluorescence ratio to pH is approximately sigmoidal, and the ratio values generated by the ImageJ analysis can be converted to pH using a generalized logistic or sigmoid function or by linear or cubic spline interpolation.
8. To measure cytoplasm, draw a line across the cytoplasm and right-click to “measure pH”.

## Representative Results

**Figure 1** presents snapshots of neutrophils from different origins to demonstrate varying phagosomal environments. To ease quantitative analysis, it is important to seed the wells with an appropriate number of cells: too many will cause the cells to layer over each other, making it difficult to view enclosed phagosomes accurately; too few will, of course, provide fewer results, particularly as not every neutrophil will phagocytose. **Figure 2** is an image that is over-saturated; this can be assessed by splitting the image between its two channels (using the microscope software recommended in the Materials List or an equivalent) — red dots show where maximum fluorescence has been detected. This can be countered by reducing the intensity of the laser. Calibration curves using the various buffer systems are shown in **Figure 3**, adapted from Levine *et al.*^5^. The error bars show that there is some variation in fluorescence between readings. **Figure 4** gives an example of how the data for phagosomal pH and area could be presented. This approach allows each individual measurement to be displayed with an over-laying boxplot. However, the data could also be displayed in a histogram bar chart.


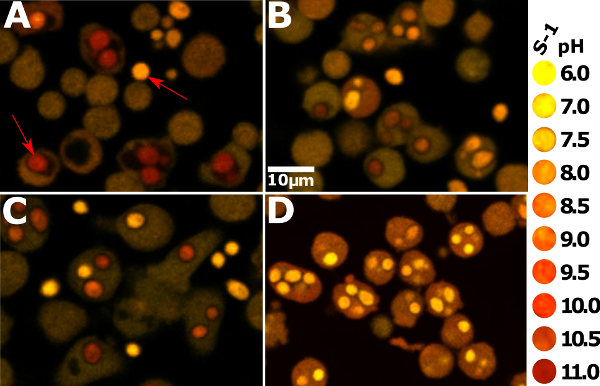
**Figure 1: Appropriate snapshot images of neutrophils from humans and mice. **To the far right is a qualitative visual key of the approximate color of the S-1-stained phagosomes corresponding to the pH. The yellow color indicates more acidity, while red indicates more alkalinity. (**A**) shows *Hvcn1^-/-^* mouse bone marrow neutrophils 20 min after phagocytosis. The phagosomes appear very red, alkaline, and swollen. The red arrow in the bottom right part of the image points to an intracellular *Candida*, while the arrow in the top left points to an extracellular particle. (**B**) shows wildtype mouse bone marrow neutrophils that have ingested *Candida*; they are much less alkaline than *Hvcn1^-/-^* cells. (**C**) shows human peripheral blood neutrophils at the same point after phagocytosis. They appear slightly more alkaline than the mouse wildtype cells, but the phagosomes are still not as large and red as the *Hcvn1^-/-^* cells. (**D**) shows human neutrophils that have phagocytosed *Candida* in the presence of 5 µM diphenylene iodonium (DPI). All the phagosomes are very acidic, with a pH of 6 or less; the drug inhibits the NADPH oxidase, so there is no compensatory ion movement. The protons released from the acidic granules that fuse to the phagosome cause the acidic pH^20^, and increased recruitment of the V-ATPase to the phagosomal membrane upon treatment with DPI^9^. The cytoplasm also appears more alkaline compared to the cells from the other conditions. Please click here to view a larger version of this figure.


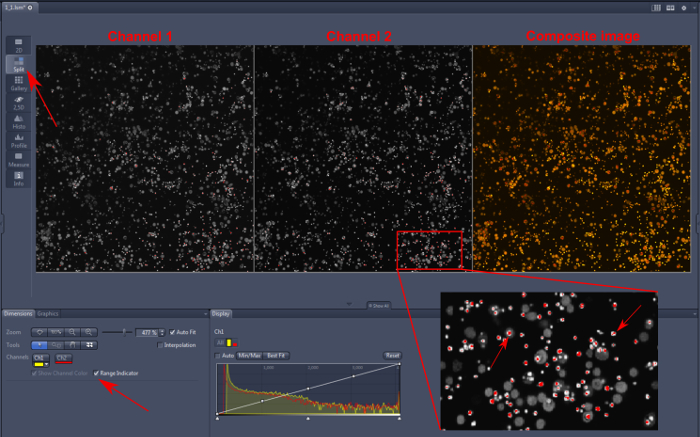
**Figure 2: Over-saturation of* Hvcn1^-/-^* mouse bone marrow neutrophils. **It is important to exclude from the analysis images in which the fluorescence data are over-saturated. As described in section 5.3, the composite image is split into two images (top left, red arrow) with both channels presented individually. In this software, range indicator is checked on (bottom left, red arrow), then any pixels which are over-saturated are bright red. There is over-saturation present in both channels (1 and 2). A magnification of some cells and extracellular *Candida* is shown at the bottom right, with arrows pointing to affected areas. Analyzing these points would lead to a false ratiometric measurement. Please click here to view a larger version of this figure.


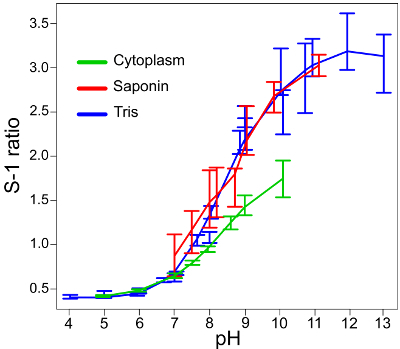
**Figure 3: Standard calibration curves for the conversion of S-1 fluorescence ratios to pH measurements. **The standard curves for *Candida* alone in the different buffers (labeled as "Tris") and when the cells are permeabilized with saponin ("Saponin") are very similar, showing that the S-1 reading inside and outside the cell (phagosome and extracellular medium) are comparable. The error bars represent the mean ± SD. The S-1 ratio/pH curve is shortened when tested in the cytoplasm ("Cytoplasm"), which should be taken into consideration in the image analysis. This figure has been modified from Levine *et al.*^5^. Please click here to view a larger version of this figure.


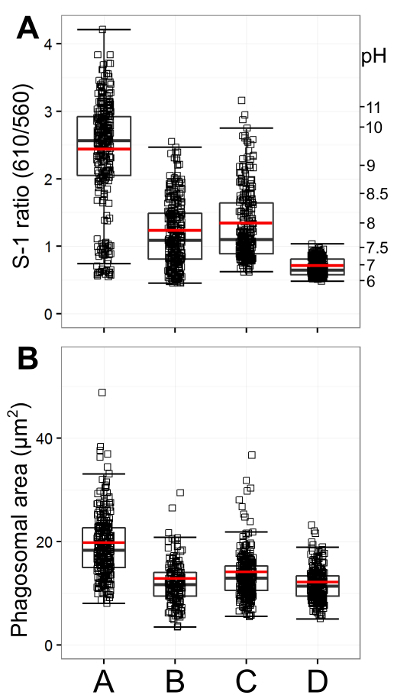
**Figure 4: Quantification of phagosomal pH and area. **This figure presents an example of data presentation. The graphs were generated using the programming software R. A: shows phagosomal ratio and corresponding pH for A (*Hvcn1^-/-^* mouse bone marrow neutrophils), B: wildtype mouse bone marrow neutrophils, C: human peripheral blood neutrophils, and D: human neutrophils with 5 µM DPI n = 3/300. Individual measurements are shown as small squares, with an overlaying boxplot with median and interquartile range. A red bar represents the mean. As seen in the images in **Figure 1**, *Hvcn1^-/-^* cells have very alkaline phagosomes in comparison to wildtype mouse and human neutrophils. They also have a bigger phagosomal area (**Figure 4B**, n = 3/300). Human neutrophil phagosomes are slightly more alkaline and larger than wildtype mouse neutrophils, while human neutrophils incubated with DPI have very acidic and small phagosomes. Please click here to view a larger version of this figure.

**Table:** 

**Balanced salt solution (BSS) buffer**
NaCl	156 mM		
KCl	3 mM		
MgSO_4_	2 mM		
KH_2_PO_4_	1.25 mM		
CaCl_2_	2 mM		
Glucose	10 mM		
Hepes	10 mM		
pH 7.4 with NaOH or HCl
**YPD broth**
YPD broth	50 g		
Distilled water	1 L		
Autoclave at 121 °C for 15 min
For YPD agar: before autoclaving add 15 g/L agar
**10% Dextran solution**
Dextran clinical grade		50 g	
NaCl		4.5 g	
Distilled water		500 mL	
Add to glass bottle and autoclave at 121 °C for 15 min
**2x Saline solution**
NaCl		18 g	
Distilled water		1 L	
Add to glass bottle and autoclave at 121 °C for 15 min
**10% Saponin stock**
BSS buffer		50 mL	
Saponin		5 g	
Heat BSS buffer to 37 °C, add saponin and mix.
Add 0.1% sodium azide as preservative, store mixture at 4 °C.
**Calibration buffers**
**Candida standard curve**
First make up 0.15 M stock of each buffer
Make up 0.15 M NaCl solution
15 mL final volume: 5 mL 0.15 M of desired buffer solution + 10 mL 0.15 M NaCl solution
pH 3	100 mM NaCl	50 mM glycine	
pH 4	100 mM NaCl	50 mM acetate	
pH 5	100 mM NaCl	50 mM acetate	
pH 6	100 mM NaCl	50 mM acetate	
pH 7	100 mM NaCl	50 mM Tris	
pH 8	100 mM NaCl	50 mM Tris	
pH 9	100 mM NaCl	50 mM Tris	
pH 10	100 mM NaCl	50 mM glycine	
pH 11	100 mM NaCl	50 mM phosphate	
pH 12	100 mM NaCl	50 mM phosphate	
pH 13	100 mM NaCl	50 mM phosphate	
**Cytosolic standard curve**
Use previously made 0.15 M stock buffer solutions
Make up 0.15 M KCl solution
15 mL final volume: 5 mL 0.15 M of desired buffer + 10 mL 0.15 M KCl solution
10 mM stock of nigericin in ethanol, add 15 µL to each final volume solution
pH 3	100 mM KCl	50 mM glycine	10 µM nigericin
pH 4	100 mM KCl	50 mM acetate	10 µM nigericin
pH 5	100 mM KCl	50 mM acetate	10 µM nigericin
pH 6	100 mM KCl	50 mM acetate	10 µM nigericin
pH 7	100 mM KCl	50 mM Tris	10 µM nigericin
pH 8	100 mM KCl	50 mM Tris	10 µM nigericin
pH 9	100 mM KCl	50 mM Tris	10 µM nigericin
pH 10	100 mM KCl	50 mM glycine	10 µM nigericin
pH 11	100 mM KCl	50 mM phosphate	10 µM nigericin
pH 12	100 mM KCl	50 mM phosphate	10 µM nigericin
pH 13	100 mM KCl	50 mM phosphate	10 µM nigericin
pH all calibration solutions with either HCl or NaOH

**Table 1: Composition of buffers. **This table describes the appropriate compositions of the different buffers used in the protocol.

**Supplementary code file. **This file contains a number of macros, written by A. P. Levine, which are necessary for image analysis. The authors would be happy to try to address any queries associated with using this code. Please click here to download this file.

## Discussion

Once the appropriate reagents, microscope settings, and calibration experiments are set up, this method is relatively simple to perform. The critical steps include: labeling the *Candida* with S-1 to ensure that there is no overloading of the dye, calibrating, and analyzing the image.

S-1 is a reagent suited to more alkaline pH environments, which is particularly important for neutrophils^21^ but limits its use in certain cell types. For more acidic environments, such as macrophage phagosomes, SNARF-4, or S-4, is more suitable because of its lower pKa^22^. Moreover, for more accurate cytoplasmic readings, it is better to use S-4, as the standard curve for S-1 shows that fluorescence ratios begin to plateau below pH 6 (**Figure 3**). Other dyes, such as 2',7'-Bis-(2-Carboxyethyl)-5-(and-6)-Carboxyfluorescein (BCECF) or pHrodo Red may also be more suitable in a context that is expected to be acidic. Yet the cytoplasm staining is still necessary for correct identification of the phagosomes containing *Candida*.

An important feature of a phagosomal pH indicator is that it is not irreversibly altered by the reactive phagosomal environment. S-1 seems to be resistant to the neutrophil milieu. This is shown by Levine *et al.*^5^ (see Supplementary Video 4 of reference^5^) which demonstrate the phagocytosis and subsequent release of an S-1-labeled *Candida *particle by an *Hvcn1^-/-^* neutrophil. When phagocytosed, the particle turns from yellow/orange to red (neutral to alkaline pH), but when the particle is released by the neutrophil, it returns to its original color.

It is important to mention some of the limitations associated with using S-1. The fact that this dye has two emission spectra allowing ratiometric measurement is an advantage, but specialist equipment is needed to acquire images; the microscope used for the experiments must be able to record two images simultaneously or with an insignificant time delay. The authors assume that the researcher attempting this protocol has experience using confocal microscopy, or has access to a trained professional. We cannot list all the specific microscope parameters as they will differ for each microscope and need to be optimized by the researcher. The acetoxymethyl ester conjugated to S-1 that allows the dye to diffuse into the cell cytoplasm is degraded by non-specific esterases in the cell cytoplasm to form the fluorescent molecule. Esterases, such as alkaline phosphatase, are present in human serum and fetal bovine serum, which are used to supplement cell culture media. Accordingly, the medium in which the cells are loaded with S-1-AM (section 4.5) must not contain serum. This may prove challenging if using cells that require a more nutrient-rich medium to sustain them than the balanced salt solution used throughout this protocol. Similarly, other fluorescent medium components, such as phenol red, may interfere with S-1 measurements.

The error bars in **Figure 3** indicate that there is some variation in the ratio measurements at each pH. A suitable number of repeats of each experiment (at least n = 3) and as many individual measurements in each single experiment are needed to overcome the inter-vacuolar variation. It is thus advisable to measure the pH of at least 100 phagosomes for each condition and as many phagosomal areas that appear to contain only one *Candida*. The phagosomes to be measured for quantitation should be those that have completely engulfed a *Candida* particle (*i.e.,* those completely surrounded by cytoplasm). To mitigate against unintentional biases in the selection of cells/phagosomes for quantitation, all analyses should be performed while blind to the experimental conditions.

Here, we describe the isolation of neutrophils by dextran sedimentation of whole blood followed by centrifugation of the plasma layer through a density gradient. We use this technique as it quickly and efficiently produces a pure (>95%) population of neutrophils, although there are other methods available, such as whole blood centrifugation through other density gradient formulas or negative selection of neutrophils using specialist kits with antibodies or magnetic beads. However, the latter can be prohibitively expensive for most groups who isolate neutrophils routinely. In addition, we use the anticoagulant heparin in the blood-collecting tube, whereas other researchers may be more accustomed to using ethylenediaminetetraacetic acid (EDTA) or acid citrate sodium (ACD). As there are many different methods to choose from, it is up to the personal preference of the researcher.

Furthermore, when isolating and manipulating neutrophils they should be handled with some care to avoid excessive activation. Precautionary steps include: only using plastic ware, no glass; filter-sterilize all buffers to remove any contaminating endotoxin; when spinning neutrophils make sure the centrifuge is well balanced to avoid excessive vibrations; limit as much as possible the time neutrophils remain in a pellet after centrifugation; do not maintain neutrophils in solution of more than 5 x 10^6^/mL; and perform the experiment as soon as possible after isolation.

This method can be adapted to measure changes in the pH and phagosomal area over time by using a heated stage set to 37 °C on the microscope and taking snapshots once every 30-60 s from the same position, as described for the calibration steps. It could also theoretically be adapted for higher-throughput experiments, such as in 96-well plates, and for flow cytometry experiments, where S-1 can be used as a pH indicator^23^. However, in these settings, the emphasis on individual cell activity is replaced by a more global effect on the cell population.

This method aims to provide a relatively simple experimental setup upon which individual researchers can adapt to suit their area of interest. Researchers may want to explore neutrophil phagosomal pH and area whilst also measuring movement of other ions, for example, intracellular calcium concentration. There are several fluorescent Ca^2+^ indicators readily available for confocal microscopy, such as Indo-1, which also has dual emission spectra at 400 and 475 nm^24^. These emission wavelengths do not overlap with S-1 emission spectra, but the excitation wavelength is at the ultraviolet (UV) end of the spectrum, which can be damaging to cells, and a UV laser is not commonplace on all microscopes. A comprehensive review of the different indicators to measure intracellular calcium flux is covered by Takahashi *et al.*^25^ and Hillson *et al.*^26^.

In conclusion, there are other methods available to measure phagosomal pH using different fluorescent dyes as Nunes *et al*. have demonstrated^13^ as well as other groups^27^,^28^. Other researchers have also used S-1 to measure cytosolic pH^29^ or phagosomal pH^14^. However, this protocol is unique in measuring simultaneously the pH of both cytosol and phagosome, which provides the opportunity to observe changes in phagosomal cross-sectional area and to distinguish between wildtype and *Hvcn1^-/-^* mouse neutrophils, and human neutrophils with and without a working NADPH oxidase.

## Disclosures

The authors declare that they have no competing financial interests.
